# Supplementary material for: Novel mouse models based on intersectional genetics to identify and characterize plasmacytoid dendritic cells
Source: Nat Immunol. 2023 Mar 16;24(4):714–28. doi: 10.1038/s41590-023-01454-9 (PMC10063451; doi:10.1038/s41590-023-01454-9)
Supplement: Supplementary file 1 — Supplementary Information [file 41590_2023_1454_MOESM1_ESM.pdf]

nature immunology

Technical Report https://doi.org/10.1038/s41590-023-01454-9

Novel mouse models based on intersectional genetics to identify and characterize plasmacytoid dendritic cells

In the format provided by the authors and unedited

Table of content

Supplementary Fig. 1

Supplementary Fig. 2

Description of Supplementary Table.

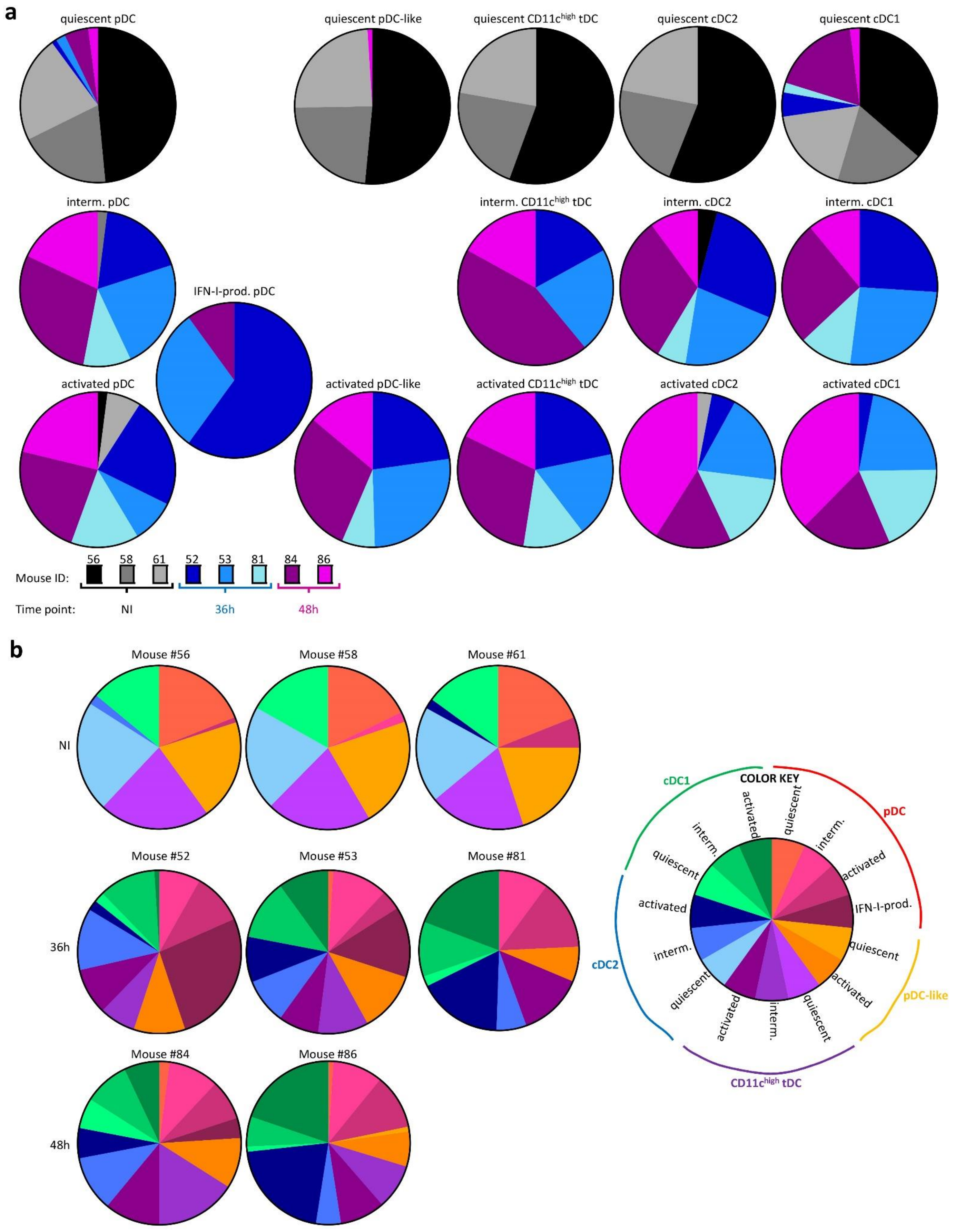

Supplementary Fig. 1. Analysis of scRNA-seq data for individual mice. a, Pie charts showing the contribution of each mouse (color code) to each of the combinations of cell type and activation state (individual pie charts) as identified in Fig. 8a. **b**, Pie charts showing the fraction of each combination of cell type and activation state (color code, as in Fig. 8a) for all the annotated cells of each mouse (individual pie charts).

Supplementary Figure 2. Strategy and parameters used for the computational analyses of the FB5P-seq single cell RNA sequencing dataset.

	Function / aim	analysis step	cells of uninfected mice only, starting #of cells: 384	whole dataset: cells of uninfected & infected mice, starting #of cells: 1343
Seurat analysis (Step#1)	Seurat version	pre-processing, normalization, dimensionality reduction, clustering, identification of marker genes	3.2.0	3.2.0
	genes expressed in at least		3 cells	7 cells
	cells expressing at least #of genes		600 genes	600 genes
	cells expressing at maximum #of genes		5000 genes	5000 genes
	cells expressing at maximum % of mitochondrial genes		<median + 3 MAD (median absolute deviation)	<median + 3 MAD
	{remaining cells/ genes}		345 cells / 12381 genes	1132 cells / 13326 genes
	NormalizeData		normalization.method = "LogNormalize", scale.factor = 1e6	normalization.method = "LogNormalize", scale.factor = 1e6
	FindVariableFeatures		selection.method = "vst", mean.function = ExpMean, dispersion.function = LogVMR, num.bin = 20, binning.method = "equal_width", nfeatures = 1000	selection.method = "vst", mean.function = ExpMean, dispersion.function = LogVMR, num.bin = 20, binning.method = "equal_width", nfeatures = 1000
	RunPCA		30 pcs	30 pcs
	FindNeighbors		5 dims, k.param=5, compute.SNN=TRUE, force.recalc=TRUE	11 dims, k.param=7, compute.SNN=TRUE, force.recalc=TRUE
	FindClusters		resolution=0.2, random.seed=0, algorithm=1	resolution=0.6, random.seed=0, algorithm=1
	RunUMAP		umap.method="uwot", 5 dims, seed.use=10, n.components=2, n.neighbors = 4, spread=1	umap.method="uwot", 9 dims, seed.use=10, n.components=2, n.neighbors = 4L, spread=1
	FindMarkers		test.use = "bimod"	test.use = "bimod"
	clusters of contaminating cells	removal of contaminants	NA	clusters 6, 9, 10, 12
	{remaining cells/ genes}		345 cells / 12381 genes	951 cells / 13326 genes
Seurat analysis (Step#2)	FindVariableFeatures	re-analysis of dataset after contaminant removal	NA	selection.method = "vst", mean.function = ExpMean, dispersion.function = LogVMR, num.bin = 20, binning.method = "equal_width", nfeatures = 2000
	RunPCA		NA	30 pcs
	FindNeighbors		NA	9 dims, k.param=9, compute.SNN=TRUE, force.recalc=TRUE
	FindClusters		NA	resolution=0.7, random.seed=0, algorithm=1
	RunUMAP		NA	umap.method="uwot", 9 dims, seed.use=10, n.components=2, n.neighbors = 4L, spread=1
Rphenograph analysis	removal of cells lacking index sorting information	integration of index sorting information and clustering	2 cells (plate12_BC12 and plate13_BC23)	3 cells (plate4_BC22, plate12_BC12 and plate13_BC23)
	{remaining cells with transcript and protein level information}		343 cells	948 cells
	Rphenograph		k = 20	k = 50
CMAP analysis	signatures used	cell type identification based on gene expression	tDC, cDC2, cDC1, pDC, pDC_vs_pDClike (external signatures generated from public data from PMIDs: 26903243, 29925996)	unambiguous relative DC_type-specific signatures, from the analysis of the cells from uninfected mice
	{#cells identified as bona fide pDC, pDC-like, tDC, cDC1, cDC2}		205 cells (Extended Data Fig. 6f); 103 pDC, 23 cDC2, 34 cDC1, 26 pDC-like, 19 tDC; identified based on combined analysis of Seurat and Rphenograph clusters (Extended Data Fig. 6d), refined by selecting cells on cMap scores[footnote_1] (Extended Data Fig. 6e)	851 cells (Fig. 4e); 310 pDC, 103 cDC2, 122 cDC1, 167 pDC-like, 149 tDC; identified based on combined analysis of Seurat and Rphenograph clusters (Extended Data Fig. 7e), and globally confirmed by the CMAP analysis (Extended Data Fig. 7f)
Seurat analysis (Step#3)	FindMarkers	adj. P-value<0.05 in both cases	test.use = "wilcox" (generation of unambiguous relative DC_type-specific signatures)	test.use = "wilcox" (generation of DEGs between activation states of a given cell type)

[footnote_1] Precise rules used to assign a DC_type to each cell.

*for pDC: (i) belonging to Seurat∩Rphenograph clusters I∩c or I∩e or I∩f, and (ii) cMAP scores obeying the following rules, AND(sgCMAP_pDC>0; sgCMAP_pDC_vs_pDClike>0; sgCMAP_tDC≤0; sgCMAP_cDC2≤0; sgCMAP_cDC1≤0).

*for pDC-like: (i) belonging to Seurat∩Rphenograph clusters II∩g, and (ii) cMAP scores obeying the following rules, AND(sgCMAP_pDC_vs_pDClike<0; sgCMAP_cDC2≤0; sgCMAP_cDC1≤0).

*for tDC: (i) belonging to Seurat∩Rphenograph clusters III∩a or V∩a, and (ii) cMAP scores obeying the following rules, AND(sgCMAP_tDC>0; sgCMAP_cDC2≤0; sgCMAP_cDC1≤0; sgCMAP_pDC≤0).

*for cDC2 (i) belonging to Seurat∩Rphenograph clusters III∩b or VI∩b, and (ii) cMAP scores obeying the following rules, AND(sgCMAP_cDC2>0; sgCMAP_cDC1≤0; sgCMAP_pDC≤0; sgCMAP_tDC≤0; sgCMAP_pDC_vs_pDClike≤0).

*for cDC1 (i) belonging to Seurat∩Rphenograph clusters IV∩d, and (ii) cMAP scores obeying the following rules, AND(sgCMAP_cDC1>0; sgCMAP_cDC2≤0; sgCMAP_pDC≤0; sgCMAP_tDC≤0; sgCMAP_pDC_vs_pDClike≤0).

Supplementary Fig 2. Strategy and parameters used for the computational analyses of the FB5P-seq single cell RNA sequencing dataset.

Description of Supplementary Table. The first spreadsheet gives the absolute numbers of DC types in the spleen early during MCMV infection. These data are derived from the spectral flow cytometry experiments. For each spleen, the percentages of each DC type within the live cell spectral flow cytometry gate was retrieved from the analysis illustrated in Extended Data Fig. 5 and multiplied by the absolute number of viable cells. Statistical analyses were performed with unpaired t test. The second spreadsheet gives the antibody panels used for the immunohistofluorescence (IHF) analyses, for each of the corresponding figure. The subsequent spreadsheets give the metadata for the single cell RNA sequencing experiment. Specifically, the third sheet lists the names of each of the following sheets and describes their content. The fourth sheet indicates, for each cell, the experimental condition ("timepoint"), the identity of the mouse it was isolated from ("mouse_ID"), its initial sorting phenotype, the results of the quality controls, its belonging to Seurat or Rphenograph clusters for the different analyses performed, its final assignment to a DC type and activation state, and its fluorescence intensity for the cell surface markers used for sorting. Gene markers for Seurat clusters, and the lists of genes differentially expressed between Seurat clusters or sub-clusters, according to the different bioinformatics analyses performed, are given in the following spreadsheets, as are the signatures used for the CMAP analyses, as listed and described in the third sheet.
