# Supplementary material for: Novel mouse models based on intersectional genetics to identify and characterize plasmacytoid dendritic cells
Source: Nat Immunol. 2023 Mar 16;24(4):714–28. doi: 10.1038/s41590-023-01454-9 (PMC10063451; doi:10.1038/s41590-023-01454-9)
Supplement: Supplementary file 2 — Reporting Summary [file 41590_2023_1454_MOESM2_ESM.pdf]

nature portfolio

Corresponding author(s): Michael Valente, Elena Tomasello and Marc Dalod

Last updated by author(s): Jan 26, 2023

Reporting Summary

Nature Portfolio wishes to improve the reproducibility of the work that we publish. This form provides structure for consistency and transparency in reporting. For further information on Nature Portfolio policies, see our Editorial Policies and the Editorial Policy Checklist.

Statistics

For all statistical analyses, confirm that the following items are present in the figure legend, table legend, main text, or Methods section.

n/a	Confirmed	
☐	☒	The exact sample size (*n*) for each experimental group/condition, given as a discrete number and unit of measurement
☐	☒	A statement on whether measurements were taken from distinct samples or whether the same sample was measured repeatedly
☐	☒	The statistical test(s) used AND whether they are one- or two-sided<br>*Only common tests should be described solely by name; describe more complex techniques in the Methods section.*
☒	☐	A description of all covariates tested
☒	☐	A description of any assumptions or corrections, such as tests of normality and adjustment for multiple comparisons
☐	☒	A full description of the statistical parameters including central tendency (e.g. means) or other basic estimates (e.g. regression coefficient) AND variation (e.g. standard deviation) or associated estimates of uncertainty (e.g. confidence intervals)
☐	☒	For null hypothesis testing, the test statistic (e.g. *F*, *t*, *r*) with confidence intervals, effect sizes, degrees of freedom and *P* value noted<br>*Give P values as exact values whenever suitable.*
☒	☐	For Bayesian analysis, information on the choice of priors and Markov chain Monte Carlo settings
☒	☐	For hierarchical and complex designs, identification of the appropriate level for tests and full reporting of outcomes
☐	☒	Estimates of effect sizes (e.g. Cohen's *d*, Pearson's *r*), indicating how they were calculated

Our web collection on statistics for biologists contains articles on many of the points above.

Software and code

Policy information about availability of computer code

Data collection	The RNA Seq data (FB5P technology) were generated using an Illumina NextSeq2000 platform, with 100-cycles P2 flow cells.
Data analysis	Conventional flow cytometry data were acquired using BD Diva v9.0. Spectral flow cytometry data were acquired with SpectroFlo 3.0.1. Flow Cytometry data were analyzed using FlowJo v10.8.1 (Treestar) and OMIQ (app.OMIQ.ai).<br>GraphPad Prism (8.1.2) was used for graphical and statistical analyses.<br>Images were processed and analyzed using ImageJ (1.52p).<br>FB5P sequencing data were aligned and mapped to reference genome using STAR (v2.5.3a) and HTSeqCount (v0.9.1) and processed to generate a single-cell UMI counts matrix (Attaf et al. Front Immunol 2020 11;216). The counts matrix was loaded to R (v.4.0.3), and Seurat (v3.2.0) was used for downstream analyses.

For manuscripts utilizing custom algorithms or software that are central to the research but not yet described in published literature, software must be made available to editors and reviewers. We strongly encourage code deposition in a community repository (e.g. GitHub). See the Nature Portfolio guidelines for submitting code & software for further information.

Data

Policy information about availability of data

All manuscripts must include a data availability statement. This statement should provide the following information, where applicable:

- Accession codes, unique identifiers, or web links for publicly available datasets
- A description of any restrictions on data availability
- For clinical datasets or third party data, please ensure that the statement adheres to our policy

The datasets generated during and/or analysed during the current study are available in the GEO repository, under accession numbers GSE76132 (PMID: 26903243) and GSE196720 (this study). All other data generated or analysed during this study are included in this published article (and its supplementary information files).

Field-specific reporting

Please select the one below that is the best fit for your research. If you are not sure, read the appropriate sections before making your selection.

☒ Life sciences ☐ Behavioural & social sciences ☐ Ecological, evolutionary & environmental sciences

For a reference copy of the document with all sections, see nature.com/documents/nr-reporting-summary-flat.pdf

Life sciences study design

All studies must disclose on these points even when the disclosure is negative.

Sample size	No statistical methods were used to pre-determine sample sizes but our sample sizes are similar to those reported in previous publications (Tomasello et al. EMBO J 2018, PMID: 30131424; Abbas et al. Nat Immunol 2020, PMID: 32690951).
Data exclusions	No animals or data points were excluded from the analyses
Replication	All experiments except scRNAseq data were reproduced at least twice. The number of experiments have been specified in each legend of figure. scRNAseq data were generated from 3 uninfected mice, 3 MCMV-infected mice for 36h and 2 infected mice for 48h, with 3 independent sorts performed with 2 or 3 animals each time (sorts for mice #56, 58 on 2020/11/03, for #52, 53 and 61 on 2020/12/17, for #81, 84 and 86 on 2021/02/11); sorting plates were frozen until all samples had been collected, and all libraries generated and sequenced simultaneously to avoid eventual batch effects. All attempts at replication were successful.
Randomization	No randomization was performed in this study. It was not necessary since mice were matched in age and gender between experimental groups and comparisons were made across cell types in the same mice or between infection time points for the same mouse strain. Hence, there were no confounding covariates in our analyses.
Blinding	No blinding was performed in this study. As we performed quantitative measurements with the use of computational analyses, the use of blinding was not required in the present study.

Reporting for specific materials, systems and methods

We require information from authors about some types of materials, experimental systems and methods used in many studies. Here, indicate whether each material, system or method listed is relevant to your study. If you are not sure if a list item applies to your research, read the appropriate section before selecting a response.

Materials & experimental systems

n/a	Involved in the study	
☐	☒	Antibodies
☒	☐	Eukaryotic cell lines
☒	☐	Palaeontology and archaeology
☐	☒	Animals and other organisms
☒	☐	Human research participants
☒	☐	Clinical data
☒	☐	Dual use research of concern

Methods

n/a	Involved in the study	
☒	☐	ChIP-seq
☐	☒	Flow cytometry
☒	☐	MRI-based neuroimaging

Antibodies

Antibodies used	anti-B220 Alexa Fluor 594 clone RA3-6B2 biolegend cat# 103254 ; RRID:AB_2563229 1/400<br>anti-B220 Alexa Fluor 647 clone RA3-6B2 biolegend cat#103229; RR1D:AB_492875 1/400<br>anti-B220 APC-Fire810 clone RA3-6B2 biolegend cat# 103278; RRID: AB_2860603 1/400<br>anti-B220 PE-cy7 clone RA3-6B2 biolegend cat# 103222 ; RRID:AB_313005 1/400

anti-B220 PerCP-Cy5.5 clone RA3-6B2 biolegend cat# 103236 ; RRID:AB_893354 1/200
anti-APC biotin clone APC003 biolegend cat# 408004 ; RRID:AB_345360 1/200
anti-BST2 BV650 clone 927 biolegend cat# 127019 ; RRID:AB_2562477 1/200
anti-BST2 BV786 clone 927 BD biosciences cat# 747603 ; RRID:AB_2744171 1/200
anti-BST2 biotin clone eBio927 Thermofisher cat# 13-3172-82 ; RRID:AB_763415 1/200
anti-BST2 purified clone 120G8 Dendritics cat#DDX0390; RRID:AB_DDX0390 1/100
anti-CCR9 PE-Cy7 clone CW-1.2 biolegend cat# 128712 ; RRID:AB_10933082 1/400
anti-CD115 BV421 clone AFS98 biolegend cat# 135513 ; RRID:AB_2562667 1/200
anti-CD115 BV711 clone AFS98 biolegend cat# 135515 ; RRID:AB_2562679 1/200
anti-CD117 BV650 clone 2B8 BD biosciences cat# 563399 ; RRID:AB_2738183 1/800
anti-CD127 BV510 clone SB/199 BD biosciences cat#563353 ; RRID:AB_2738153 1/100
anti-CD11b BUV395 clone M1/70 BD biosciences cat# 565976 ; RRID:AB_27382 1/400
anti-CD11b BV750 clone M1/70 biolegend cat# 101267;RRID:AB_2810328 1/600
anti-CD11c BUV395 clone N418 BD biosciences cat#744180;RRID:AB_2742045 1/200
anti-CD11c BUV737 clone N418 BD biosciences cat# 749039 ; RRID:AB_2873433 1/400
anti-CD11c BV421 clone HL3 BD biosciences cat# 562782 ; RRID:AB_2737789 1/400
anti-CD11c BV785 clone N418 biolegend cat# 117336 ; RRID:AB_2565268 1/200
anti-CD11c purified clone N418 biolegend cat# 117302 ; RRID:AB_313771 1/100
anti-CD135 APC clone A2F10.1 BD biosciences cat# 560718 ; RRID:AB_1727425 1/200
anti-CD169 biotin clone MOMA-1 abcam cat# ab51814 1/500
anti-CD169 Alexa Fluor 647 clone 3D6.112 biolegend cat# 142408 ; RRID:AB_2563621 1/500
anti-CD19 clone 1D3 Alexa Fluor 700 BD biosciences cat# 557958 ; RRID:AB_396958 1/200
anti-CD19 clone 1D3 BV510 BD biosciences cat# 562956; RRID:AB_2737915 1/300
anti-CD26 BUV737 clone H194-112 BD biosciences cat# 741729; RRID: AB_2871099 1/200
anti-CD3 purified clone 145-2C11 BD biosciences cat# 550275 ; RRID:AB_393572 1/300
anti-CD3 Alexa Fluor 700 clone eBio500A2 eBioscience cat# 56-0033-82 ; RRID:AB_837094 1/200
anti-CD3 Alexa Fluor 488 clone 17A2 biolegend cat# 100210; RRID:AB_389301 1/300
anti-CD3 BUV805 clone 17A2 BD biosciences cat#741982; RRID; AB_2871285 1/200
anti-CD3 BV510 clone 17A2 BD biosciences cat#740147 ; RRID:AB_2739902 1/300
anti-CD3 EF450 clone 17A2 Thermofisher cat# 48-0032-82 ; RRID:AB_1272193 1/300
anti-CD45 PerCP clone 30F11 biolegend cat# 103130; RRID:AB_893343 1/200
anti-CD45.2 BUV395 clone 104 BD biosciences cat# 564616 ; RRID:AB_2738867 1/300
anti-CD45.2 BUV737 clone 104 BD biosciences cat# 612778 ; RRID:AB_2870107 1/300
anti-CD64 BV711 Alexa Fluor 647 clone X54-5/7.1 biolegend cat# 139322; RRID:AB_2566560 1/200
anti-CD8a APC-cy7 clone 53-6.7 biolegend cat# 100714 ; RRID:AB_312753 1/200
anti-CD8a Pacific Orange clone 5H10 eBioscience cat# MCD0830; RRID:AB_10376311 1/200
anti-CD88 BUV661 clone 20/70 BD biosciences cat#750080; RRID:AB_2874295 1/400
anti-CX3CR1 BV650 clone SA011F11 biolegend cat#149033; RRID:AB_2565999 1/200
anti-CX3CR1 BV711 clone SA011F11 biolegend cat# 149031 ; RRID:AB_2565939 1/100
anti-EpCAM EF450 clone G8.8 Thermofisher cat# 48-5791-82 ; RRID:AB_10717090 1/200
anti-F4/80 BB700 clone T45-2342 BD biosciences cat#746070; RRID:AB_2743450 1/400
anti-F4/80 biotin clone BM8 biolegend cat# 123106 ; RRID:AB_893501 1/200
anti-GFP Alexa Fluor 488 polyclonal Thermofisher cat# A-21311 ; RRID:AB_221477 1/200
anti-I-A/I-E BUV496 clone M5/114.15.2 BD biosciences cat#750281; RRID:AB_2874472 1/400
anti-IE1 purified Capri clone IE1.01 cat# HR-MCMV-12 1/1000
anti-IgD Spark NIR 685 clone 11-26c.2a biolegend cat# 405750; RRID:AB_2888693 1/1000
anti-IgM BV711 clone RMM-1 biolegend cat# 406539; RRID:AB_2814386 1/200
anti-Ly6C APC-Cy7 clone AL-21 BD Biosciences cat#560596 ; RRID:AB_1727555 1/400
anti-Ly6C BV510 clone HK1.4 biolegend cat# 128033;RRID:AB_2562351 1/800
anti-Ly6D EF450 clone 49-H4 thermofisher cat# 48-5974-80 ; RRID:AB_2574089 1/400
anti-Ly6D FITC clone 49-H4 biolegend cat# 138606 ; RRID:AB_11203888 1/400
anti-Ly6G BB700 clone 1A8 BD Biosciences cat#566453; RRID:AB_2739730 1/400
anti-Ly6G BV510 clone 1A8 BD Biosciences cat#740157 ; RRID: AB_2739910 1/300
anti-Ly6G clone 1A8Alexa Fluor 700 biolegend cat# 561236 ; RRID:AB_10611860 1/200
anti-mcherry purified polyclonal Rockland cat# 600-401-P16 1/500
anti-NK1.1 Alexa Fluor 700 clone PK136 BD biosciences cat# 553162 ; RRID:AB_394674 1/200
anti-NK1.1 APC-Cy7 clone PK136 biolegend cat# 156505; RRID:AB_2876525 1/200
anti-NK1.1 BV510 clone PK136 biolegend cat# 108738 ; RRID:AB_2562217 1/300
anti-RFP purified polyclonal Rockland cat# 600-401-379 1/500
anti-SiglecH BV421 clone 440c BD biosciences cat# 566581 ; RRID:AB_2739747 1/400
anti-SiglecH FITC clone 551 biolegend cat# 129604 ; RRID:AB_1227761 1/200
anti SiglecH PerCP-cy5.5 clone 551 biolegend cat# 129614 ; RRID:AB_10643995 1/200
anti SiglecH PerCP-eFluor710 clone eBio440c eBiosciences cat#46-0333-82; RRID:AB_1834443 1/100
anti-SiglecH APC clone 551 biolegend cat#129612 ; RRID:AB_10641134 1/400
anti-XCR1 BV421 clone ZET biolegend cat#148216; RRID:AB_2565230 1/400
anti-XCR1 BV650 clone ZET biolegend cat#148220 ; RRID:AB_2566410 1/800
streptavidin APC BD biosciences cat# 349024 1/400
streptavidin EF450 Thermofisher cat# 129614 1/400
streptavidin Alexa Fluor 633 Thermofisher cat# S-21375 1/400
Donkey anti-rabbit IgG (H+L) Alexa Fluor 488 poyclonal Jackson Immunoresearch cat# 711-545-152 1/200
Donkey anti-rabbit IgG (H+L) Alexa Fluor 594 poyclonal Jackson Immunoresearch cat# 127-585-152 1/500
Donkey anti-rabbit IgG (H+L) Alexa Fluor 647 poyclonal Thermofisher cat# A-31573 1/500
Goat anti-Hamster IgG (H+L) Cyanine 3 poyclonal Jackson Immunoresearch cat# 127-165-160 1/500
Goat anti-Hamster IgG (H+L) Rhodamine Red X poyclonal Jackson Immunoresearch cat# 127-295-159 1/500
Goat anti-Hamster IgG (H+L) Alexa Fluor 594 poyclonal Thermofisher cat# A-21113 1/500
Goat anti-Mouse IgG2a Alexa Fluor 633 polyclonal Thermofisher cat# A-21136 1/2500

Goat anti-Rat Alexa Fluor 546 polyclonal Thermofisher cat# A-11081 1/350

Validation

All antibodies were validated by the manufacturer, for the species and the specific application for which they were used in the study (flow cytometry, immunohistofluorescence). Each antibody has been previously titrated for optimal performance in the used assay.

Animals and other organisms

Policy information about studies involving animals; ARRIVE guidelines recommended for reporting animal research

Laboratory animals

C57BL/6J M/F 6-16 weeks
Siglech-iCre;Rosa26-LSL-RFP M/F 8-16 weeks
pDC-Tom (Siglech-iCre;Pacsin1-LSL-tdT) M/F 8-16 weeks
SCRIPT (Siglech-iCre;Pacsin1-LSL-tdT;Ifnb1-eYFP) M/F8-16 weeks
ZeST (Siglech-iCre;Pacsin1-LSL-tdT;Zbtb46-eGFP) M/F 8-16 weeks
Mice were housed under 12h dark/12h light cycle, with a temperature range of 20-22°C and a humidity range of 40-70%.

Wild animals

This study did not use wild animals

Field-collected samples

This study did not involve field-collected samples

Ethics oversight

All animal experiments were performed in accordance with national and international laws for laboratory animal welfare and experimentation (EEC Council Directive 2010/63/EU, September 2010). Protocols were approved by the Marseille Ethical Committee for Animal Experimentation (registered by the Comité National de Réflexion Ethique sur l'Expérimentation Animale under no. 14; APAFIS#1212-2015072117438525 v5 and APAFIS#21626-2019072606014177 v4).

Note that full information on the approval of the study protocol must also be provided in the manuscript.

Flow Cytometry

Plots

Confirm that:

☒ The axis labels state the marker and fluorochrome used (e.g. CD4-FITC).

☒ The axis scales are clearly visible. Include numbers along axes only for bottom left plot of group (a 'group' is an analysis of identical markers).

☒ All plots are contour plots with outliers or pseudocolor plots.

☒ A numerical value for number of cells or percentage (with statistics) is provided.

Methodology

Sample preparation

Samples originated from mouse organs.
For flow cytometry, spleens or lymph nodes were harvested and submitted to enzymatic digestion for 25 minutes at 37°C with Collagenase IV (Wortington biochemicals) and DNAse I (Roche Diagnostics). Organs were then mechanically digested and passed over 100µm cell strainers (Corning). Red blood cells were then lysed by using RBC lysis buffer (Life Technologies) for spleen and bone marrow cell preparation. Livers were harvested, minced and submitted to enzymatic digestion, as for the spleen. Liver pieces were then crushed and cell suspension obtained was washed 2 times with PBS 1x, before performing a 80/40 Percoll gradient. Cells isolated from the middle ring of the gradient were washed once with PBS 1x, then used for flow cytometry. Small intestines were harvested, opened longitudinally, then cut into 1mm pieces. Pieces were washed entensively with PBS 1x, then incubated 3 times at 37°C upon shaking (200 rpm) with PBS 1X containing 2% Fetal Calf Serum (FCS) and 5mM ethylenediamine tetraacetic acid (EDTA). At the end of each incubation, supernatants were collected and centrifuged. Pelleted cells, mainly IntraEpithelial Cell (IEL), from the three incubations were pooled together and submitted to a 67/44 Percoll gradient. Cells isolated from the middle ring of the gradient were washed once with PBS 1x, then used for flow cytometry.
For microscopy, organs were fixed with Antigen Fix (Diapath) for 2 hours for the small intestine, colon, lymph nodes or 4 hours for the spleen at 4°C, and then washed several times in PB (0,025 M NaH2PO4 and 0,1 M Na2HPO4). Organs were then immerged in a solution of 30% sucrose O/N at 4°C. Organs were then embedded in OCT (Sakura), snap frozen and stored at -80°C.

Instrument

BD Fortessa X-20, BD ARIA 3, Cytek Aurora

Software

BD DIVAv9.0, FlowJo v10.8.1, SpectroFlo 3.0.1, OMIQ

Cell population abundance

The abundance of the population was low since it was single cell sorting (scRNAseq)

Gating strategy

The gating strategies have been provided in the Extended Data Figures.
Figure 1= CD64-neg/CD45-pos/live, then for neutrophils: Ly6G-pos; for B cells: CD19-pos/Ly6G-neg/CD3-neg; for NK and T cells: Ly6G-neg and then CD3-neg/NK1.1-pos versus CD3-pos/NK1.1-neg, respectively; for pDC: Ly6G-neg/CD3-neg/NK1.1-neg/CD11c-low/BST2-pos, and for cDC: Ly6G-neg/CD3-neg/NK1.1-neg/CD11c-pos/Bst2-neg and CD8a-pos (for cDC1) vs CD11b-pos (for cDC2).

Figure 2a= for CMP, CDP and MDP CD11c-neg/MHCII-neg/FLT3-pos followed by CD117-high/CD115-neg vs CD117-pos/CD115-pos vs CD117-neg/CD115-pos, respectively; for preDC: CD11c-pos/MHCII-neg/FLT3-pos/CD115-neg-to-pos followed by segregation according to Ly6C vs SiglecH expression; for pDC: Lin-neg/B220-pos/CD11c-pos/MHC-II-pos/SiglecH-pos/Bst2-pos; for CD11c+ pre-pDC: Lin-neg/B220-pos/CD11c-pos/MHC-II-neg/FLT3-pos/CD115-neg-to-pos/SiglecH-pos/Bst2-pos.

Figure 2b= for LP: MHCII-neg/CD11c-neg/FLT3-pos/B220-neg/CD117-neg/CD115-neg/CD127-pos, followed by segregation according to Ly6D vs SiglecH expression.

Extended Figure 5a= CD88-neg/Ly6G-neg, then for B cells: CD19pos IgMpos; for NK, NKT and T cells: CD19-neg/IgM-neg followed by segregation according to CD3 vs NK1.1 expression; for pDC vs tDC: CD3-neg/NK1.1-neg followed by XCR1-neg/CD11b-neg/(CD11c-pos or BST2-pos) and then Ly6D-pos for pDC versus Ly6D-neg/ CX3CR1-pos/CD26-pos for tDC further split according to Ly6C expression; for cMo: CD3-neg/NK1.1-neg/CD11b-pos/Ly6C-pos; for cDC: CD3-neg/NK1.1-neg/CD11c-pos/MHC-II-pos followed by XCR1-pos (for cDC1) vs CD11b-pos (for cDC2).

☒ Tick this box to confirm that a figure exemplifying the gating strategy is provided in the Supplementary Information.
